# Neurotrophic Factors in the Treatment of Acute Brain Hypoxia Secondary to Cardiac Arrest: a Case Report

**DOI:** 10.25122/jml-2019-1007

**Published:** 2019

**Authors:** D Węgrzyn, A Kutwin-Chojnacka, J Bilski, K Mroszczyk, K Węgrzyn

**Affiliations:** 1. Anesthesiology and Intensive Care Department, Maria Sklodowska-Curie’s District Hospital, Skarzysko Kamienna, Poland; 2. Danylo Halytskyi, Lviv National Medical University, Lviv, Ukraine

**Keywords:** hypoxic brain injury, neuroprotection, neuroplasticity

## Abstract

Finding neuroprotective agents to counteract the deleterious effects of hypoxia on neuronal cells successfully is one of the most critical targets of clinical research since preclinical studies have identified potential neuroprotective strategies. In clinical practice, amantadine and piracetam are used with reasonable success. We present the cases of three patients with acute brain hypoxia secondary to cardiac arrest, to whom Cerebrolysin was added to the standard neuroprotective treatment regimen, leading to a notable improvement in functional outcome.

## Introduction

This article highlights three case reports that describe our experience with adjunctive therapy for acute brain hypoxia after cardiac arrest, using cerebrolysin - a multimodal agent has been suggested to promote neuroprotection and neuroregeneration after stroke [1,2] and traumatic brain injury [3]. Under hypoxic conditions, some mechanisms are at work resulting in a deadly cascade of toxic events which cause immediate and delayed tissue damage in the brain. Furthermore, recent clinical studies have demonstrated a synergistic effect between cerebrolysin treatment and an early, intensive rehabilitation program in patients suffering from ischemic stroke [4]. In our institution, we have been using neuroprotective agents with different mechanisms of action for many years, including amantadine and piracetam. We have recently added cerebrolysin to our standard treatment regimen in patients with acute brain hypoxia after cardiac arrest, for which we report the present series of outcomes.

## Case 1

A 59-year-old female patient was brought to the Emergency Room (ER) by the emergency medical services after a cardiac arrest at home and successful resuscitation. The emergency medical service intubated the patient who received bag-valve-mask ventilation and passive oxygen therapy. Administered medications were adrenaline and atropine. The electrocardiography (ECG) performed in the ER showed a discrete ST elevation in the II, III, and aVF leads.

The patient was admitted to the Intensive Care Unit (ICU) in critical condition, deeply unconscious with a Glasgow Coma Scale (GCS) of 3 points, myosis and delayed pupillary light reaction. Respiratory function was insufficient, demanding mechanical ventilation. The patient was unstable hemodynamically, with a heart rate of 110 bpm while the blood pressure initially was140/80 mmHg and then 90/60 mmHg. Electrocardiographic manifestations of acute myocardial ischemia were present. The laboratory tests demonstrated high D-dimer concentrations as well as elevated creatinine and potassium level, so ion-exchange resin was administered. Treatment with 200mg/day amantadine, 30ml/day cerebrolysin, 12g/day piracetam was administered, while antithrombotic, sedative, and bronchodilators agents were used if needed. The result of the head CT on day one showed a focus of encephalomalacia in the left caudate nucleus. Apart from that, the cortex had no other signs of malacia. The ventricular system was in the midsagittal plane with normal width and symmetry. No signs of intracranial bleeding or fractures were detected, and passive rehabilitation started in the ICU on day 3. Treatment regimen with cerebrolysin (30 ml/day), piracetam and amantadine was maintained until the end of hospitalization in the ICU. On day 12, the patient was taken off the ventilator and extubated.

The patient stayed in the ICU for 13 days and subsequently moved to the neurological department in a stable and much-improved condition with verbal communication. In the neurological unit, the patient stayed for 17 days, and treatment with 30 ml of cerebrolysin per day was continued. At discharge, the patient presented with improved neurological state and psychomotor functions, sphincter control, and independent feeding. The patient walked independently with minor support. Bilateral cortical blindness with light sensitivity and color recognition were still present.

## Case 2

A 42-year-old female patient was admitted to the ICU after a sudden cardiac arrest due to ventricular fibrillation, being resuscitated before arriving at the hospital. The anamnesis showed mitral valve regurgitation and ventricular arrhythmia. The general condition was severe: the patient was unconscious, scored 4 points on the GCS, had symmetric pupils with delayed light reaction. Spastic tetraplegia and decerebrate posture were present, and the Babinski sign was absent bilaterally. The patient was mechanically ventilated and had an irregular cardiac rhythm of 120 bpm with a blood pressure of 100/60 mmHg and the presence of numerous ventricular extrasystoles. Laboratory investigations showed increased Troponin I, CK-MB, D-dimers, transaminases, CRP and leukocytosis. Head CT showed features of cerebral edema. Mechanically ventilation was kept for 7 days, followed by a tracheostomy (Griggs method), right pleural catheterization (after iatrogenic pneumothorax) for 13 days, antibiotic treatment (at first empiric, later targeted), anticoagulants, antiplatelets, medication for heart disease, diuretics, sedative drugs, 12mg/day piracetam for 8 days and then 2x12mg/day, 200mg/day amantadine, 30ml/day cerebrolysin. Bedside rehabilitation was initiated on day 7.

The patient was transferred to the cardiology ward after 23 days of treatment in the ICU in good general condition. She was conscious, and verbal communication was possible most of the time. The GCS score was 13, without any motor deficit and with adequate circulatory and respiratory functions.

## Case 3

A 65-year-old male patient was admitted to ICU from the ER after sudden cardiac arrest and prolonged resuscitation. Respiratory and circulatory functions were insufficient. The medical history showed an acute coronary disease (NSTEMI) in April 2007 with angioplasty surgery of left circumflex coronary artery and hypertension.

The patient was intubated in the emergency room. The patient arrived in the ICU being deeply unconscious, scored 3 on the GCS 3, had myosis and pupillary light reaction. Additionally, sinus tachycardia with 150 beats per min was noticed. The patient was connected to life support and mechanically ventilated. ECG showed features of acute coronary disease, high values of CK-MB as well as troponin I. Brain CT showed bilaterally inhomogeneous hypodense areas in the frontal lobes, no indication of pre-existing diseases and traumas, as well as signs of cerebral edema. There was no evidence of bleeding or traumatic skeletal changes. Segmental thickening of frontal sinuses mucus was present, and left-sided curvature of the nasal sinus with the presence of bone spurs was also observed. Mechanical ventilation was followed by administration of neurorestorative and neuroprotective treatment with 30ml/day of cerebrolysin, 12g/day of piracetam, 200mg/day of amantadine and sedation with midazolam as needed. Medication for heart disease, diuretics, antiplatelets, anticoagulants, antibiotics, amino acids, and crystalloids were also administered. The patient was extubated on day 7 with an improved condition; he was able to follow commands and to answer basic questions.

On day 8, another sudden cardiac arrest occurred. Resuscitation was performed and resulted in the recovery of the cardiac function. The patient was intubated and connected to life support again. Due to prolonged intubation, a tracheotomy was performed. On the last two days of hospitalization in the ICU, piracetam was discontinued. Cerebrolysin (30ml) and amantadine (200mg) were administered during the whole course of hospitalization. After 33 days, the patient was transferred to the neurology ward with an improved overall condition, with adequate respiratory and circulatory functions. He was conscious, with global aphasia, inability to follow commands, periodical psychomotor hyperactivity, tetraparesis and Babinski sign present on the right side. Physical rehabilitation was carried out. He was hospitalized in the neurology ward for 15 days. Cerebrolysin (30ml/day) was administered throughout the whole duration of stay in the neurology ward.

At discharge, the patient was conscious, and verbal communication was possible with occasional non-logic periods. The GCS score was 15, with no paresis and adequate circulatory and respiratory functions.

## Conclusion

These cases illustrate the effects we typically achieve in our clinic with our new standard treatment regimen, which includes cerebrolysin, piracetam, and amantadine. Our previous neuroprotective regimen typically resulted in a reasonably good clinical outcome in most cases; however, after adding cerebrolysin, we were able to see such a spectacular recovery in patients with severe brain hypoxia who typically have an abysmal clinical prognosis. For a reliable confirmation of our observations, long-term and well-planned controlled studies are required. Furthermore, a head-to-head comparison of the individual components of our treatment regimen would help to assess the individual effects and to determine any synergistic effect by combining neuroprotective and neurorestorative treatments.

## Conflict of Interest

The authors confirm that there are no conflicts of interest.

## References

[1] Guekht A. (2017). Safety and efficacy of Cerebrolysin in motor function recovery after stroke: a meta-analysis of the CARS trials. *Neurol Sci*.

[2] Muresanu D. F., Strilciuc S., Stan A. (2019). Current Drug Treatment of Acute Ischemic Stroke: Challenges and Opportunities. *CNS Drugs*.

[3] Poon W. (2019). Safety and efficacy of Cerebrolysin in acute brain injury and neurorecovery: CAPTAIN I-a randomized, placebo-controlled, double-blind, Asian-Pacific trial. *Neurol. Sci.*.

[4] Muresanu D. F. (2016). Cerebrolysin and Recovery After Stroke (CARS): A Randomized, Placebo-Controlled, Double-Blind, Multicenter Trial. *Stroke*.

